# Therapeutic effect of all-trans-retinoic acid (at-RA) on an autoimmune nephritis experimental model: role of the VLA-4 integrin

**DOI:** 10.1186/1471-2369-8-3

**Published:** 2007-01-24

**Authors:** María M Escribese, Elisa Conde, Ana Martín, David Sáenz-Morales, David Sancho, Guillermo Pérez de Lema, Javier Lucio-Cazaña, Francisco Sánchez-Madrid, María L García-Bermejo, Francisco M Mampaso

**Affiliations:** 1 Department of Pathology, Hospital Ramón y Cajal, Universidad de Alcalá, Madrid, Spain; 2 Department of Biology, Universidad SEK, Segovia, Spain; 3 Department of Immunology, Hospital de la Princesa, Universidad Autónoma de Madrid, Madrid, Spain; 4 Medizinische Poliklinik der Ludwig Maximillians-Universität, Munich, Germany; 5 Department of Physiology, Universidad de Alcalá, Madrid, Spain; 6 Dpt. of Microbiology, Mount Sinai School of Medicine, New York (NY), USA

## Abstract

**Background:**

Mercuric chloride (HgCl_2_) induces an autoimmune nephritis in the Brown Norway (BN) rats characterized by anti-glomerular basement membrane antibodies (anti-GBM Ab) deposition, proteinuria and a severe interstitial nephritis, all evident at day 13 of the disease. We assessed the effects of all-trans retinoic acid (at-RA) in this experimental model. At-RA is a vitamin A metabolite which has shown beneficial effects on several nephropathies, even though no clear targets for at-RA were provided.

**Methods:**

We separated animals in four different experimental groups (HgCl_2_, HgCl_2_+at-RA, at-RA and vehicle). From each animal we collected, at days 0 and 13, numerous biological samples: urine, to measure proteinuria by colorimetry; blood to determine VLA-4 expression by flow citometry; renal tissue to study the expression of VCAM-1 by Western blot, the presence of cellular infiltrates by immunohistochemistry, the IgG deposition by immunofluorescence, and the cytokines expression by RT-PCR. Additionally, adhesion assays to VCAM-1 were performed using K562 α4 transfectant cells. ANOVA tests were used for statistical significance estimation.

**Results:**

We found that at-RA significantly decreased the serum levels of anti-GBM and consequently its deposition along the glomerular membrane. At-RA markedly reduced proteinuria as well as the number of cellular infiltrates in the renal interstitium, the levels of TNF-α and IL-1β cytokines and VCAM-1 expression in renal tissue. Moreover, we reported here for the first time in an *in vivo *model that at-RA reduced, to basal levels, the expression of VLA-4 (α4β1) integrin induced by mercury on peripheral blood leukocytes (PBLs). In addition, using K562 α4 stable transfectant cells, we found that at-RA inhibited VLA-4 dependent cell adhesion to VCAM-1.

**Conclusion:**

Here we demonstrate a therapeutic effect of at-RA on an autoimmune experimental nephritis model in rats. We report a significant reduction of the VLA-4 integrin expression on PBLs as well as the inhibition of the VLA4/VCAM1-dependent leukocyte adhesion by at-RA treatment. Thereby we point out the VLA-4 integrin as a target for at-RA *in vivo*.

## Background

Brown Norway (BN) rats receiving sublethal doses of HgCl_2 _develop a self-limiting autoimmune syndrome characterized by the presence of an autoreactive Th2 CD4^+ ^cell subset [1-3]. This autoimmune response is accompanied by synthesis and linear anti-GBM IgG Ab deposition as well as severe proteinuria. The histological renal lesions consist of a mild and transient glomerular influx of circulating leukocytes and a severe and persistent cell infiltrate in the renal interstitium that reach the maximum at day 13 of the disease [3]

The vitamin A metabolite, at-RA, which regulates a broad range of biological processes, has been reported to be a beneficial treatment in the rat model of anti-Thy1.1 mesangioproliferative glomerulonephritis, reducing mesangial expansion and proteinuria [4] as well as in the glomerulonephritis induced by anti-GBM antibodies, ameliorating necrosis and urinary protein excretion [5]. These previous evidences of at-RA effects on experimental renal models of diseases suggest that this compound could have beneficial effects on the development of an inflammatory and autoimmune response in the kidney. Up to the moment, the mechanisms and the targets through which at-RA treatment improves renal diseases outcome remains quite unexplored.

For the development of an inflammatory response, as the one evidenced in this experimental model, it is well known that the interaction of the circulating leukocytes with the endothelium and the subsequent extravasation into tissues is necessary [6], as well as T-B cells interaction, in the development of an autoimmune response, [7] also present in this experimental model. In this regard, previous results from our laboratory described that VLA-4 integrin is critical for leukocyte migration from blood to renal tissue in HgCl_2_-induced nephritis [8,9]. In addition, VLA-4 not only mediates the adhesion and trans-endothelial migration of leukocytes, but also provides co-stimulatory signals that contribute to the activation of T lymphocytes and participates in the immunological synapse between antigen presenting cells and T cells [10], thus underscoring the important role of VLA-4 in this experimental model.

On the other hand, it has been described that at-RA modulates the expression and function of β2 integrins in primary human monocytes [11]. In addition, it has been reported that at-RA abolished *in vitro *α4 integrin dependent rolling in the acute promyelocytic leukaemia cell line NB-4 [12]. However, there are not previous evidences regarding to the modulation of VLA-4 integrin expression and function by at-RA *in vivo*.

Conversely, cytokines such as IL-1β and TNF-α stimulate the expression of adhesion molecules required for leukocyte adhesion and migration in endothelial cells [13]. Besides, it has been reported that at-RA affects VCAM-1 expression in a dermal microvascular endothelium cell line by modulating the expression of TNF-α [14].

We have therefore studied, using an experimental *in vivo *model of autoimmune nephritis in rats, the effect of at-RA in the outcome of this renal disease. We reported herein that at-RA significantly reduces VLA-4 expression and function as well as VCAM-1 expression, both mediating, among others, the beneficial effect of at-RA in this nephropathy. Moreover, the present work identifies for the first time the VLA-4 integrin as a target for at-RA *in vivo*.

## Methods

### Animals

Male BN rats, weighting 150 to 180 g, were obtained from IFFA-CREDO (Paris, France) and from our own breeding colony, fed with standard laboratory chow *ad libitum *and given free access to water. All experimental procedures were performed according to the institutional guidelines that are in compliance with the National Institute of Health Guide for the Care Use of Laboratory animals.

### Cell culture

The human cell line K562 was grown in RPMI 1640 supplemented with 10% fetal bovine serum, 2 mM glutamine, 100 U/ml penicillin and 100 μg/ml streptomycin (Invitrogen), considered as complete medium, in a humidified atmosphere with 5% CO_2_, at 37°C. K562-α4 cells (K562 transfected with α4 cDNA) were maintained in complete medium also containing 1.5 mg/ml G418 (GIBCO) [15]. Both cell lines were treated with 50, 100 and 500 nM at-RA during 24, 48 and 72 h.

### Experimental in vivo design

Animals were divided into four different groups. Group I (n = 10) received five subcutaneous injections of HgCl_2 _(1 mg/Kg body weight) over a period of two weeks for inducing the disease and was considered as positive control group (the HgCl_2 _injections start at day 0 of the experiment). The dosage and days were chosen based on previous optimizing experiments [9]. The following two groups of rats (Groups II and III) were treated from day 0 of the experimental protocol with chow pellet supplemented with at-RA (15 mg/Kg body weight). At-RA supplemented chow pellets were prepared daily using a 1.68 mg/ml ethanol at-RA stock solution, as previously described [16], and mixed in a dark cold room with standard chow. After the ethanol evaporated, the food was given to the rats. Group II (n = 15) was formed by rats injected with HgCl_2 _and treated with at-RA (15 mg/Kg) and group III (n = 10) by rats only treated with at-RA. Finally, in group IV (n = 10) rats were injected with H_2_O adjusted at the same pH (3.8) as the HgCl_2_solution, following the same protocol of HgCl_2 _administration and this group was considered as control group.

### Proteinuria

Rats were housed in metabolic cages with free access to food and water to collect 24 h urine. Urine samples were taken at day 0 and day 13th. Protein concentration in urine was determined by Bio-Rad assay (Bio-Rad, Richmond, CA) according to the manufacturer's protocol. Samples were assayed in triplicate, and the optical density from each one was measured at 595 nm in a Titertek Multiskan Plus (Flow, Irvine, Scotland).

### Anti- GBM abs assay

Rat GBM was isolated as described [17]. Briefly, glomeruli were obtained from healthy BN rats by differential sieving and centrifugation of minced kidney cortices. The glomerular suspension was sonicated, washed and lyophilized. The GBM was digested by collagenase I (Sigma Chemical Co., St. Louis, MO) at 0.7% wt/wt at 37°C for 1 h. Anti-GBM Abs in serum were measured by ELISA, as previously described [17] using rat GBM extracted as adhesion substrate for the anti-GBM Abs. All the samples were assayed in triplicate. Samples of a serum pool from untreated BN rats and from mercury-treated BN (which were blended at day 13 of the disease) served as negative and positive controls, respectively. Results were expressed as percentage of binding relative to samples from positive control serum. Absorbance was measured at 492 nm using the Titertek Multiskan Plus.

### Kidney tissue processing

Kidneys were removed at day 13 of the disease and studies were performed on paraffin-embedded tissue and cryosections. For light microscopy, a piece of tissue was fixed in phosphate-buffered 10% formalin, and 3 μm paraffin-embedded kidney sections were stained with periodic acid-Shiff (PAS). An indirect peroxidase stained method was used to characterize CD68^+^cells (anti-rat monocyte/macrophages) and CD3^+^cells (anti-rat T lymphocytes), in the renal interstitium [8,18]. These antibodies were obtained from Serotec (Oxford, U.K). Quantification of interstitial infiltrating cells bearing positive CD68 and CD3 cell surface markers was performed by counting, in two kidney tissue sections per each rat, the total number of positive labelled cells examined in ten randomly chosen areas of interstitial infiltrates, as previously described [19]. Kidney tissue cryosections (5 μm) fixed in ether/ethanol, were used for VLA-4^+ ^cells detection with the mouse anti-human HP2/1 mAb directed towards the α4 integrin that cross-reacts with rat α4 integrin [8], following the same indirect peroxidase method as described above. Direct immunofluorescence studies were also performed on ether/ethanol fixed serial kidney tissue cryostat sections using FITC-conjugated rabbit anti-rat IgG Ab from Serotec (Oxford, U.K), as described [20]. Finally, the fluorescence intensity of IgG deposits was quantified using ScionImage software in five different fields from three independent sections in all experimental groups.

### Protein extraction and western blot analysis

Pieces of renal tissue were snap-frozen in liquid nitrogen and lysed in 100 mM phosphate (pH 7.6) buffer containing: 150 nM NaCl, 10 mM EDTA, 0.5% Triton X-100 and phosphatase (1 mM FNa, 2 mM ONa) and protease inhibitors (10 mg/ml leupeptin, 10 mg/ml aprotinin, 25 mM AEBSF). Homogenized tissue was incubated on ice for 30–60 min and lysates were centrifuged at 13000 × g for 30 min at 4°C. Supernatants were collected and protein concentration was quantified by Bradford colorimetric assay. Whole kidney lysates were separated by SDS-PAGE and transferred onto PVDF membranes (Millipore, Bedford, MA). Membranes were incubated in blocking solution for 1 hour. Anti-VCAM-1 Ab and anti-actin (Santa Cruz, CA) were diluted in Tris- Buffered saline, 0.05% Tween containing 1% BSA (Sigma) and membranes were incubated overnight at 4°C. The secondary antibodies rabbit anti-goat HRP-conjugated (Jakson ImmunoResearch, PA), diluted in the same buffer mentioned above, were incubated at room temperature for 1 h. Specific bands were visualized by ECL Western Blotting detection system (Amersham Pharmacia Biotech).

### Reverse transcriptase polymerase chain reaction (RT-PCR)

For quantitative reverse transcriptase polymerase chain reaction (RT-PCR), snap-frozen kidney tissues from all experimental groups of rats were homogenized with a Polytron^® ^(Kinematica, Littau, Switzerland) and total RNA was obtained using the Ultraspec RNA isolation system (Biotecx, Houston, Texas). 2 μg of DNaseI-treated RNA was reverse transcribed with MuLV reverse transcriptase (Roche). mRNA expression was quantified by real-time PCR following the manufacturer's instructions (Lightcycler rapid thermal cycler, Roche) using the primers specific for sequences from different exons that generated products (5'→3'): ATG CCA TCC TGC GTC TGG ACC TGG C (β-actin, sense), AGC ATT TGC GGT GCA CGA TGG C (β-actin-antisense), CAC CTC TCA AGC AGA GCA CAG (IL-1β-sense), GGG TTC CAT GGT GAA GTC AAC (IL-1β-antisense), CCA GGA GAA AGT CAG CCT CCT (TNFα-sense), TCA TAC CAG GGC TTG AGC TCA (TNFα-antisense). Results for the expression of each cytokine were normalized to expression and both cytokine and β-actin were measured in each sample.

### Flow cytometry assay

Whole-blood samples of heparinized blood (100 μl aliquots) were incubated with saturating concentrations of anti-α4 HP2/1 at 4°C for 45 min. After washing with PBS, the cells were incubated with goat anti-mouse FITC-conjugated (Jakson ImmunoResearch, PA) at 4°C for 30 min. The erythrocytes were lysed with Quicklysis, following manufacturer's instructions. Cells incubated only with the secondary Ab served as negative control for non-specific antibody binding. The samples were analyzed using FACScan cytometer (Becton Dickinson, Oxford, UK).

### Cell adhesion assay

For cell adhesion, K562-α4 transfectants were labelled with BCECF-AM (Molecular probes, Leiden, The Netherlands) for 30 min at 37°C, and some samples were also pre-incubated with anti-α4 HP2/4. Then, 5 × 10^4^/100 μl cells were added in adhesion medium (RPMI containing 0.4% BSA) to 96-well dishes (High-binding; Costar, Cambridge, MA) coated with sVCAM-1 1 μg/ml (R&D Systems). After incubation for 20 min at 37°C, unbound cells were removed by three washes with RPMI medium and adhered cells were quantified using a fluorescence analyzer (CytoFluor 2003; Millipore, Bedford, MA).

### Statistical analyses

Proteinuria and ELISA results were given as mean ± SD. The statistical analysis was performed by ANOVA analysis and a P < 0.001 was considered significant.

## Results

### Effect of at-RA on proteinuria

**Figure 1:**
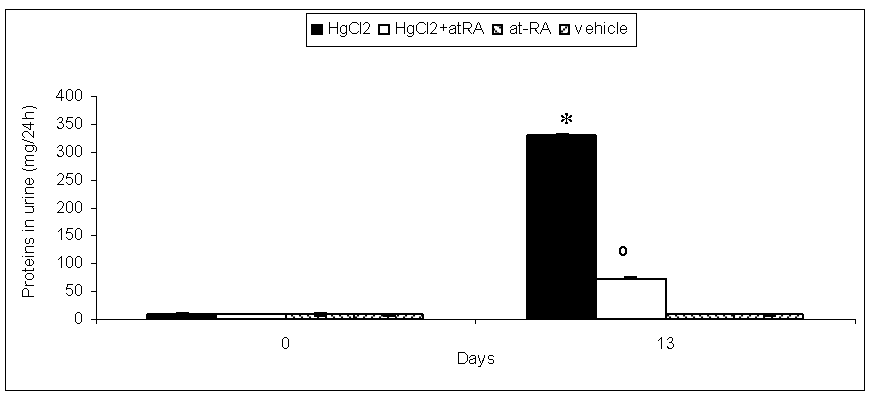
**At-RA treatment reduces proteinuria**. Protein excretion in urine from all the experimental groups, measured by colorimetric assay (Bradford Method). Results are expressed as mg of protein/24 h. Data are presented as mean ± SD; *P < 0.001 vs. vehicle group and °P < 0.001 vs. HgCl_2 _group;

To ascertain the effect of at-RA on characteristic parameters for the HgCl_2_-induced nephritis, we first evaluated the effect of at-RA on the protein excretion levels (Fig. 1). Proteinuria from HgCl_2_-injected rats (group I) reaches significant values at day 13. When HgCl_2_-injected rats were treated with at-RA, the urinary levels of protein excretion showed an 80% reduction at day 13 of the disease. Rats only treated with at-RA showed no modifications in urinary protein levels as occurred in control rats.

### Effect of at-RA on anti-GBM abs production and glomerular deposition

**Figure 2:**
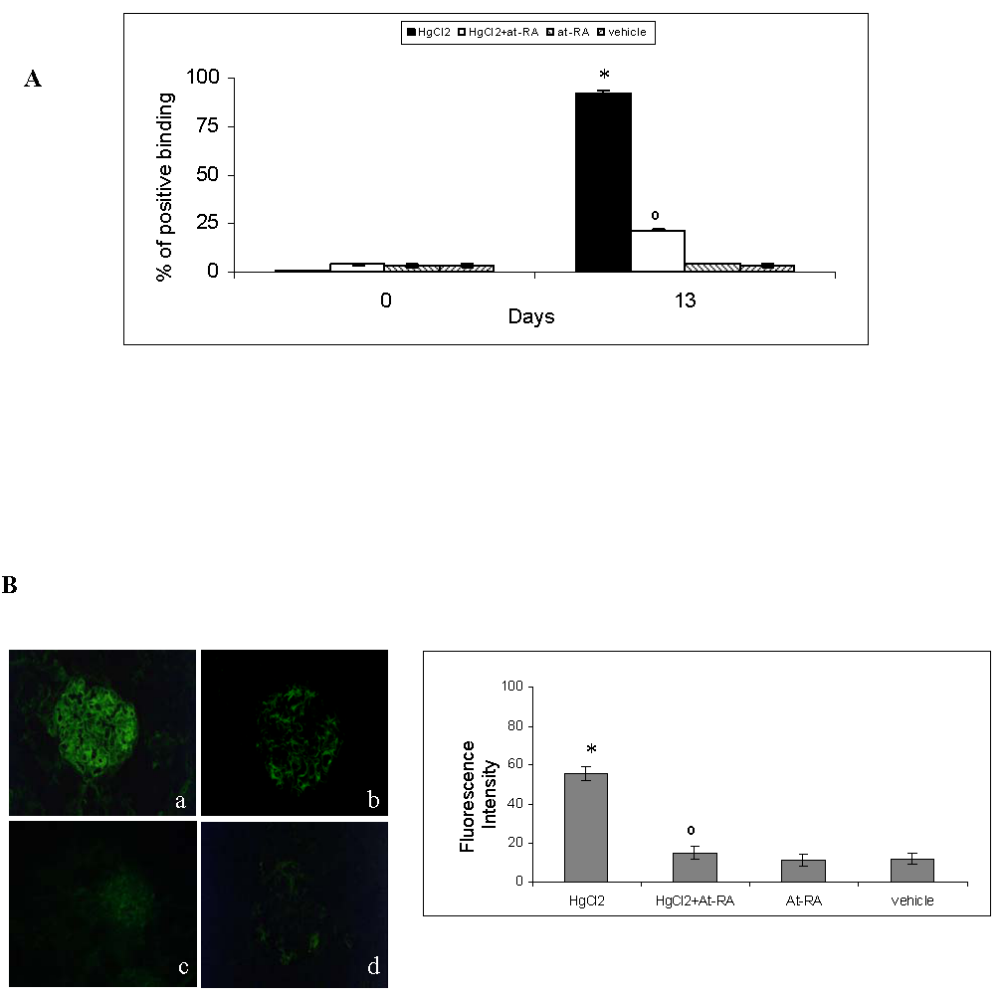
**At-RA treatment decreases anti-GBM abs production and deposition along GBM**. **A) **Serum levels of circulating anti-GBM Abs in all experimental groups measured by ELISA. Results were expressed as percentage of binding relative to samples from positive control serum and presented as Mean ± SD;* P < 0.001 vs. vehicle group and °P < 0.001 vs. HgCl_2 _group. **B) **Glomerular deposits of IgG measured by direct immunofluorescence in kidney cryosections from HgCl_2_-injected rats (a), HgCl_2_-injected rats also treated with at-RA (b), rats treated only with at-RA (c) and injected with vehicle (d). Ig G fluorescence intensity quantification is shown on the right. Data are presented as mean ± SD; *P < 0.001 vs. vehicle group and °P < 0.001 vs. HgCl_2 _group.

Next, the synthesis of anti-GBM auto-Ab was evaluated. As shown in Fig. 2A, at day 13 of the disease HgCl_2_-injected rats showed an increase of anti-GBM Ab circulating levels in the serum (group I) estimated by ELISA. In addition, a positive linear pattern of rat IgG deposits along the GBM was detected by direct immunofluorescence in this experimental group (Fig. 2B,a). Otherwise, rats that in addition to mercury administration were treated also with at-RA (group II) showed a significant decrease at day 13 in both circulating serum levels (Fig. 2A) and glomerular deposition of IgG anti-GBM Ab (Fig. 2B,b). The IgG deposition along the GBM was quantified measuring fluorescent intensity emitted by the deposited Abs. This quantification confirmed the significant reduction of anti-GBM Abs deposition in kidney sections from HgCl_2_-injected rats also treated with at-RA in comparison to sections from HgCl_2_-injected rats (Fig. 2B).

### Effect of at-RA on renal interstitial cell infiltrates

**Figure 3:**
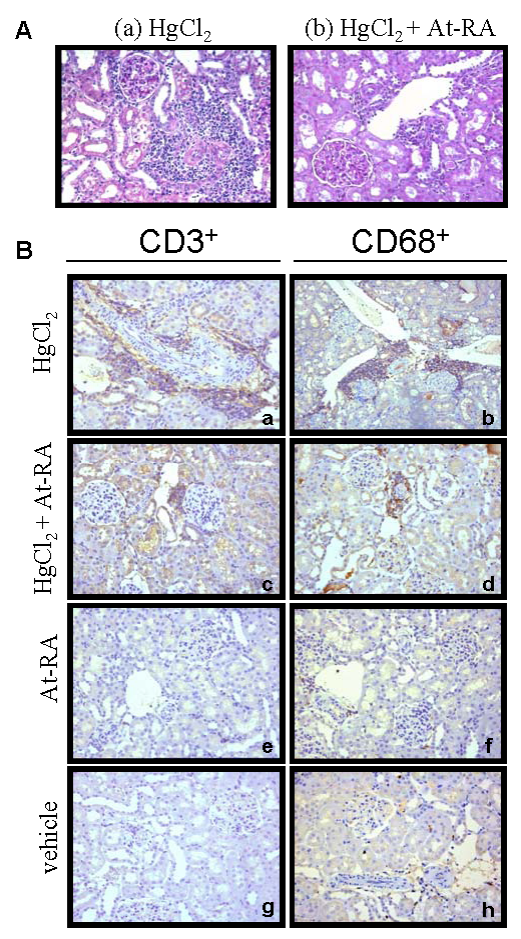
**Renal interstitial leukocyte infiltration is diminished by at-RA treatment**. **A) **Interstitial cell infiltration evaluated by light microscopy in periodic acid-Shiff (PAS)-stained renal tissue. Representative images from HgCl_2_-injected rats (a) and HgCl_2_-injected rats and also treated with at-RA 15 mg/Kg (b) are shown. **B) **Renal interstitial mononuclear cell infiltrates characterization. Immunoperoxidase staining for CD3^+ ^and CD68^+ ^cells in renal tissue at day 13 of the disease, from HgCl_2_-injected rats (a-b), from HgCl_2_-injected rats also treated with at-RA (c-d), from rats treated only with at-RA (e-f) and vehicle (g-h).

Light microscopy examination of tissue sections stained with PAS indicated that rats with HgCl_2_-induced nephritis (group I), at day 13 of the disease, exhibited a pronounced interstitial cell infiltration, preferentially located in the perivascular region of the renal interstitium (Fig. 3A,a), in comparison with those rats also treated with at-RA (group II) (Fig. 3A,b). At day 13, quantification of interstitial cell infiltrates showed a significant reduction in the number of positive stained cells for lymphohaematopoietic markers in those rats that in addition to HgCl_2 _were treated with at-RA (CD3^+ ^cells: 6 ± 0.78 cells/HPF and CD68^+ ^cells: 5.75 ± 0.75 cells/HPF) as compared with those rats injected only with HgCl_2 _(CD3^+ ^cells: 32 ± 2.08 cells/HPF and CD68^+^: cells 25.50 ± 1.66 cells/HPF), (Fig. 3B,a–d). Rats from groups III and IV showed very low number of positive stained cells in the renal interstitium (CD3^+ ^cells: 2.15 ± 0.28 cells/HPF and CD68^+ ^cells: 1.10 ± 0.14 cells/HPF and CD3^+ ^cells: 2.90 ± 0.38 cells/HPF and CD68^+ ^cells: 1 ± 0.13 cells/HPF), respectively (see, Fig. 3B,e–h). Statistical significance comparing CD3+ and CD68+ infiltrated cells quantification between group I and group II was found (p < 0.001).

### At-RA treatment reduces the expression of VCAM-1 in renal tissue. Expression of pro-inflammatory TNF-α and IL-1β cytokines

**Figure 4:**
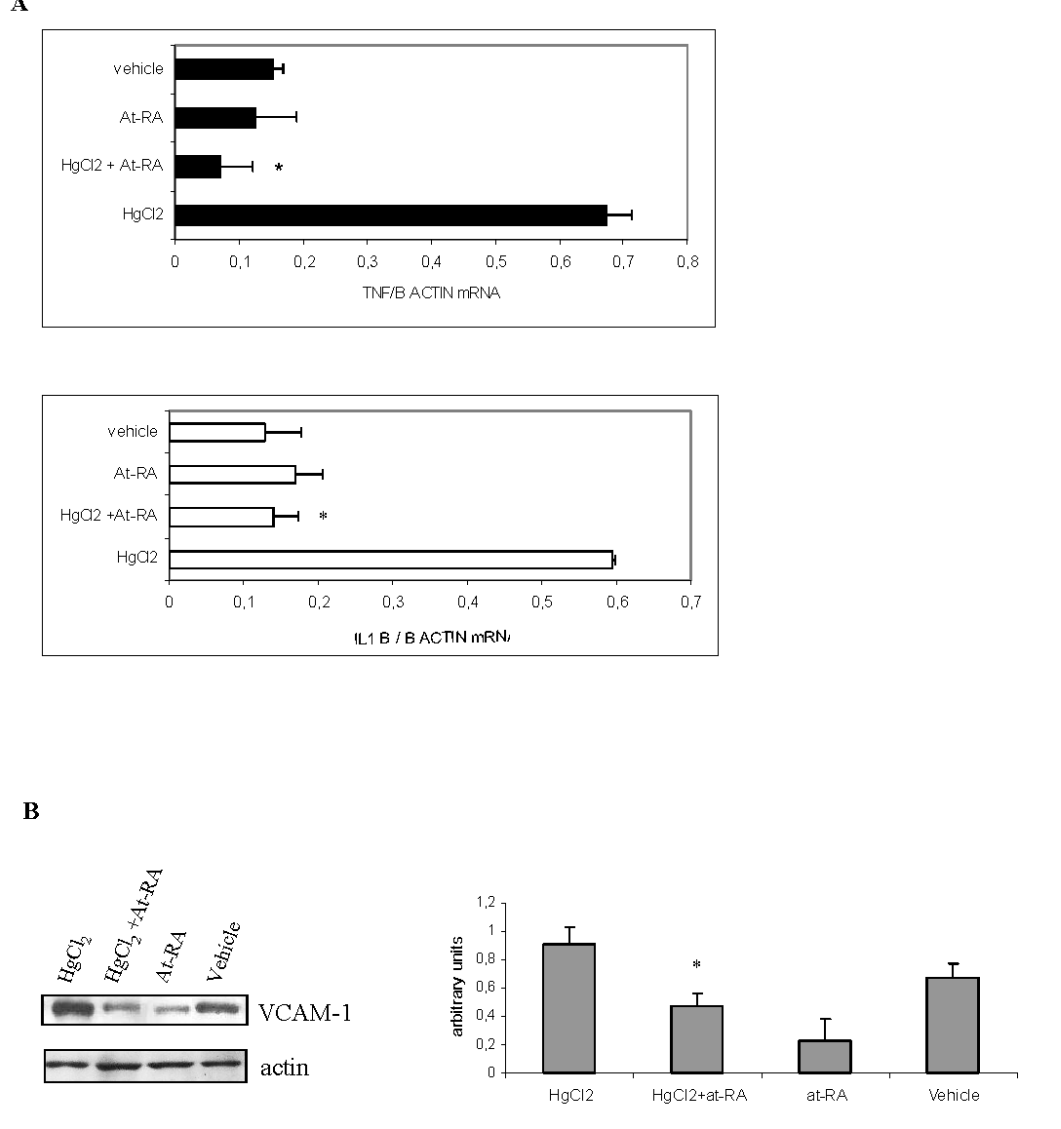
**At-RA treatment reduces proinflammatory mediators and VCAM-1 expression in renal tissue**. **A) **Proinflammatory cytokines mRNA analysis at day 13. Quantitative real-time RT-PCR of total RNA isolated from kidneys from all experimental groups. Results normalized to β-actin are expressed as mean ± SD; *P < 0.001 vs. HgCl_2_-injected rats. **B) **Expression of VCAM-1 in total renal lysates estimated by Western Blot. On the right, quantification of VCAM-1 expression is expressed as the mean ± SD of the densitometry values, relative to actin;*P < 0.001 vs. HgCl_2_-injected rats.

TNF-α and IL-1β are common mediators in inflammatory processes, and they both regulate the expression of VCAM-1. We therefore analyzed, by RT-PCR, the expression of these pro-inflammatory cytokines in total RNA extracted from kidneys at day 13 of the disease in all the experimental groups (Fig. 4A). In HgCl_2_-injected rats, the levels of TNF-α and IL1-β were up-regulated in comparison with control rats (0.67 ± 0.23 and 0.59 ± 0.03 versus 0.15 ± 0.03 and 0.12 ± 0.004). However, rats injected with HgCl_2 _and also treated with at-RA exhibited a pronounced decrease of both cytokines expression (TNF-α: 0.07 ± 0.015 and IL-1β: 0.140 ± 0.05). In addition, western blot analysis of VCAM-1 protein using total kidney lysates (Fig. 4B) showed an augmented expression of VCAM-1 at day 13 in rats only injected with HgCl_2_. Administration of at-RA to HgCl_2_-injected rats caused a significant down-regulation of VCAM-1 expression in the renal tissue. The expression of VCAM-1 from rats treated only with at-RA (group III) showed no significant differences respect to those rats injected with vehicle (group IV). All together these results indicate that at-RA treatment reduced VCAM-1 expression in HgCl_2_-injected rats and this reduction may be related to the diminished levels of TNF-α and IL-1β in the renal tissue after at-RA treatment.

### At-RA reduces the expression of VLA-4 integrin

**Figure 5:**
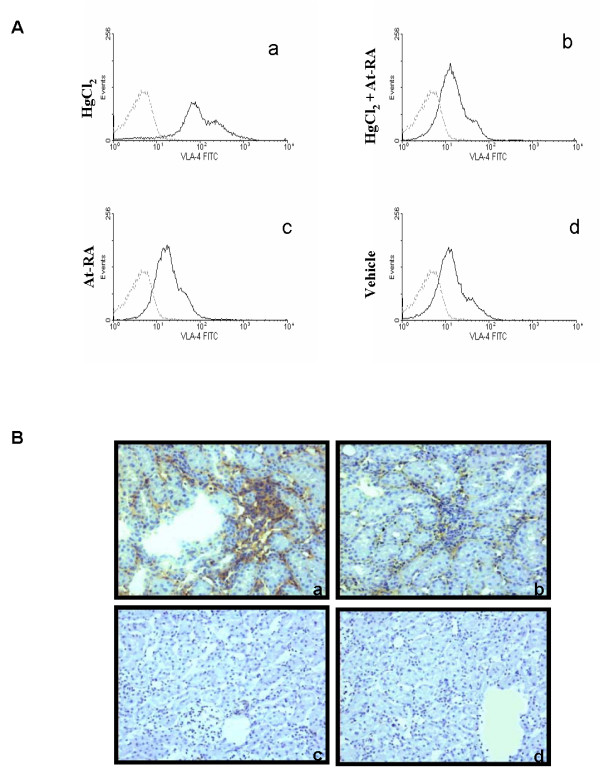
**VLA-4 integrin leukocyte expression is decreased by at-RA treatment**. **A) **VLA-4 expression in PBLs from the four experimental groups. Flow cytometry analyses were performed using anti-α4 Ab (HP2/1) (continuous line) and anti-mouse IgG Ab as a negative control (discontinuous line). PBLs from HgCl_2 _-injected rats stained with anti-α4 (a), from HgCl_2_-injected rats also treated with at-RA (b), from rats only treated with at-RA (c) and from rats injected with vehicle (d). **B) **VLA-4 expression in cellular renal infiltrates at day 13. Anti-α4 immunoperoxidase staining of renal tissue from HgCl_2_-injected rats (a), HgCl_2_-injected rats treated with at-RA (b), rats treated only with at-RA(c) and rats injected with vehicle (d)

Previous works reported that in this model of autoimmune nephritis, VLA-4/VCAM-1 is the principal adhesion pathway for circulating leukocytes extravasation into renal interstitium [8]. Therefore, we have studied the effects of at-RA on VLA-4 integrin expression. PBLs obtained at day 13 of the disease from all experimental groups were incubated with HP2/1 (anti-α4 subunit of VLA-4 integrin), and VLA-4 expression was analyzed by flow cytometry. We found that VLA-4 membrane expression on leukocytes from HgCl_2_-injected rats (group I), was significantly higher compared to that from control rats (Fig 5A,a). Interestingly, the expression of this integrin was significantly reduced in leukocytes from HgCl_2_-injected rats treated with at-RA (Fig. 5A,b) (group II) reaching basal levels. Leukocytes from rats treated with at-RA alone (group III) and control rats showed similar levels of VLA-4 expression, as shown in Fig. 5A,c and 5A,d respectively.

Additionally, the expression of VLA-4 integrin was evaluated in the infiltrated cells in the renal interstitium. About 80% of the mononuclear infiltrating cells were found positive for α4 integrin expression (Fig. 5B,a), being drastically reduced to 10 % when mercuric rats were also treated with at-RA (Fig. 5B,b). Rats from groups III and IV did not show any significant renal infiltrates.

### At-RA modulates VLA-4 adhesion to sVCAM-1

**Figure 6:**
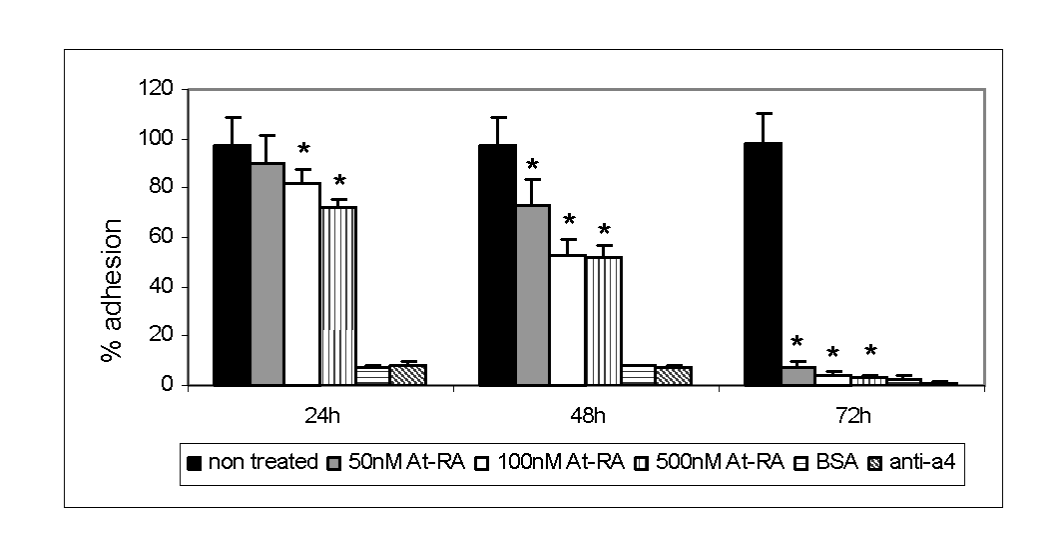
**At-RA treatment blocks VLA-4-dependent cell adhesion**. The effects of at-RA in VLA-4-dependent adhesion was estimated performing adhesion assays to VCAM-1. of K562-α4 treated with 50, 100 and 500 nM of at-RA during 24, 48 and 72 h. Cell adhesion was quantified in a fluorescence analyser previous labelling the cells with BCECF-AM. Data are presented as mean ± SD of triplicate of samples from three independent experiments. *P < 0.001 vs. non-treated K562-α4 cells.

In order to asses the effect of at-RA on VLA-4-dependent adhesion, adhesion assays to VCAM-1 were performed using K562-α4 cell treated with different at-RA doses (50, 100 and 500 nM) during 24, 48 and 72 h. Data shown in Fig. 6 demonstrate that adhesion of K562-α4 cells to VCAM-1 was inhibited by at-RA in a time and concentration dependent manner (Fig. 6).

## Discussion

In the present work, we have described the beneficial effect of at-RA in the model of autoimmune nephritis induced by HgCl_2_, and identified VLA-4 as target for at-RA in this experimental renal disease. Mercury induces in BN rats a T-dependent polyclonal B cell activation resulting in the development of an autoinmune response, with the synthesis of auto-Ab, mainly anti-GBM IgG Ab. The anti-GBM Ab was deposited along the GBM following a linear pattern, thereby altering the glomerular filtration barrier and contributing to the production of proteinuria [3] as well as to the release of pro-inflammatory mediators [1-3]. At-RA treatment has shown to be efficient in several experimental models of renal diseases [16,21-25]. In these studies, at-RA administration was associated with a reduction of inflammatory cell infiltrates as well as a decrease in the expression of proinflammatory cytokines. In our model, we have similarly demonstrated a beneficial effect of this compound in the outcome of the HgCl2-induced autoimmune nephritis, by administration of at-RA from day 0 of the experimental protocol. Moreover, we have also reported a significant effect of at-RA on the VLA-4 expression *in vivo *not described up to the moment, pointing out this integrin as a target for at-RA action.

Our results demonstrate that at-RA significantly reduces the production of circulating anti-GBM at day 13 and consequently the deposition of this auto-Ab along the GBM favoring the reduction of the proteinuria levels at day 13. The capacity of at-RA to preserve the structure and function of podocytes, involved in the maintenance of the filtration barrier, may also contribute to decrease proteinuria at day 13 [16]. In addition, preliminary results from our laboratory indicate that at-RA protects the proximal tubule epithelium integrity against oxidative injury, thus contributing to the reduction of proteinuria which is due to mercury-induced tubular toxicity (M.M. Escribese et al., unpublished observations).

Another essential feature of the inflammatory response in HgCl_2_-induced nephritis is the appearance of a massive cellular infiltrate localized in the periglomerular and perivascular zone, including macrophages (CD68+) and T lymphocytes (CD3+). The treatment with at-RA also abrogates this cell infiltration into the renal interstitium. In fact, infiltrating cells were about 5-fold lower in rats treated with at-RA than in HgCl_2_-injected rats.

Anti-α4 Ab administration has been shown to exert a protective effect against infiltration of the renal interstitium accompanied by reduction of proteinuria and of auto-Ab anti-GBM [8]. On the other hand, it has been demonstrated that VLA-4 integrin participates in the immune synapses necessary for the development of an immune response. Moreover, the blockage of VLA-4 integrin, shifts the immune response from Th2 to Th1 *in vitro *and *in vivo *in the autoimmune nephritis induced by HgCl_2 _[9,10,26]. These works point out VLA-4 integrin as an essential molecule in the development this autoimmune nephritis.

In these report, we have described a significant effect of at-RA on VLA-4 integrin expression and function. Indeed, the number of cells positive for VLA-4 in the renal interstitium was considerably lower in HgCl2-injected rats also treated with at-RA than in mercuric rats. Moreover, the expression of this integrin in PBLs from at-RA treated rats was also significantly lower than in PBLs from mercuric rats. These results clearly demonstrate that at-RA was able to reduce to basal levels the expression of VLA-4 integrin in leukocytes. The decrease in VLA-4 expression caused by at-RA could contribute to the improvement of the outcome of this autoimmune nephritis. Furthermore, our data demonstrate that at-RA has an effect on the VLA-4-dependent adhesion since the treatment of K564-α4 with at-RA reduces the capacity of these cells to adhere to VCAM-1 in a time and dose dependent manner. These *in vitro *results could explain the reduction of cellular infiltrates in renal interstitium in mercuric rats also treated with at-RA. Then, our results strongly suggest that VLA-4 integrin is an *in vivo *target for this metabolite.

Our results show, for the first time in an *in vivo *experimental model of renal disease, that at-RA reduces the plasma membrane expression of VLA-4 on PBLs from HgCl_2_-injected rats, to the basal levels found on PBLs from healthy rats. *In vitro *studies have shown that at-RA was able to modulate the expression of integrins in different cell lines as well as in primary leukocytes. Most studies have examined the effects of at-RA on β2 integrin expression [27-30]. In this regard, it has been reported that at-RA reduces the expression of all β2 integrins in primary human monocytes and induces β2 integrin expression in human monocytic cell lines THP-1 and U937 [11]. In our experimental model of autoimmune nephritis, the expression of β2 integrins such as LFA-1 was not significantly affected by at-RA treatment (data not shown), suggesting the specificity of at-RA effect on the β1 integrin, VLA-4 in our case. Regarding this, a limited number of studies had addressed the *in vitro *effects of at-AR on β1 integrins [31,32], even though it has been described that at-RA was able to significantly alter the adhesion phenotype of the acute promyelocytic leukemia cell line NB-4 under physiologic flow conditions, by regulating cell surface expression of the α4β1 integrin [12].

At-RA reduced significantly the inflammatory response developed in this nephritis model. It is well known that the increase of VCAM-1 expression in renal tissue induced by the pro-inflammatory cytokines TNF-α and IL-1β, leads to circulating leukocytes recruitment via interactions with their cognate ligands in endothelial cells [6]. In our model of mercury-induced nephritis, the levels of TNF-α and IL1-β cytokines as well as the expression of VCAM-1 molecule were significantly increased at day 13 of the disease in comparison with the controls. Pro-inflammatory cytokines are responsible of the triggering and the maintenance of a standard inflammatory response including the one developed in our experimental model [33,34]. In fact, when HgCl_2_-injected rats were treated with at-RA, the levels of both cytokines were reduced, and the expression of the endothelial adhesion molecule VCAM-1 decreased. These results suggest that the cytokine-mediated induction of VCAM-1 is prevented by at-RA treatment in our model might be through a direct action of at-RA over the VCAM-1 promoter and/or an indirect action of at-RA down-regulating the levels of proinflammatory cytokines involved on VCAM-1 expression. Further investigations to shed light into both issues are necessary.

## Conclusion

This work demonstrates that at-RA has a therapeutic effect on the HgCl_2_-induced autoimmune nephritis in BN rats, since this compound reduces significantly the inflammatory and the autoimmune responses. Our results also demonstrate that at-RA treatment reduces the membrane expression of VLA-4 on circulating PBLs, diminishes VCAM-1 expression in renal tissue and blocks VLA-4 dependent adhesion. In summary, our findings strongly suggest that VLA-4 is a target for at-RA *in vivo *and that at-RA could be considered as a therapeutic agent in those diseases where VLA-4/VCAM-1-dependent adhesion plays an essential role.

## Abbreviations

Ab = Antibody

At-RA = All trans-Retinoic Acid

BN = Brown Norway

GBM = Glomerular basal membrane

Ig G = Immunoglobulin G

IL-1β = Interleukin, 1beta

PBL = Peripheral blood leukocytes

RT-PCR = Reverse transcription-polymerase chain reaction

TNF-α = Tumor necrosis alpha

VCAM-1 = Vascular cell adhesion molecule-1

VLA-4 = Very late antigen-4

## Competing interests

The author(s) declare they have not competing interests

## Authors' contributions

MME performed most of the experiments, the statistical analyses and the writing and the editing of the manuscript. EC contributed to the performing of flow cytometry assays, ELISA and proteinuria measure. AM was responsible for immunohistochemistry assays, DSM contributed to *in vivo *experiments and manuscript editing. DS performed RT-PCRs. GPL and JLC contributed to the writing and the editing. FSM was very helpful with the experimental design, provided tools and materials and contributed to the writing and editing of the manuscript. MLGB contributed to *in vitro *experiments, coordinated studies, and was responsible for the writing and the editing of the manuscript. FM coordinated *in vivo *studies, and was responsible for the writing and the editing of the manuscript. All the authors read and approved the final manuscript.

## Pre-publication history

The pre-publication history for this paper can be accessed here:


